# Metastasis of Pregnancy-Associated Breast Cancer (Suspected to Be Hereditary Breast and Ovarian Cancer) to the Brain, Diagnosed at 18 Weeks' Gestation: A Case Report and Review of the Literature

**DOI:** 10.1155/2016/9813253

**Published:** 2016-02-11

**Authors:** Tomohiro Okuda, Sakura Yamamoto, Seiki Matsuo, Hisashi Kataoka, Jo Kitawaki

**Affiliations:** ^1^Department of Obstetrics and Gynecology, Fukuchiyama City Hospital, 231 Atsunaka-cho, Fukuchiyama, Kyoto 620-8505, Japan; ^2^Department of Obstetrics and Gynecology, Graduate School of Medical Science, Kyoto Prefectural University of Medicine, 465 Kajii-cho, Kawaramachi-Hirokoji, Kamigyo-ku, Kyoto 602-8566, Japan

## Abstract

We report a case of pregnancy-associated breast cancer with metastasis to the brain, likely resulting from hereditary breast and ovarian cancer (HBOC). A 35-year-old woman (gravida 2, para 0-1-0-1) underwent a right mastectomy and right axillary dissection after a cesarean section at 30 years of age; her mother died at 47 years of age due to breast cancer. Histopathological examination indicated an invasive ductal carcinoma with triple-negative cancer (cancer stage 2B [pT3N0M0]). The patient refused adjuvant therapy because of the risk of infertility. After 4 years, she became pregnant naturally. At 18 weeks' gestation, she experienced aphasia and dyslexia due to brain metastasis. The pregnancy was terminated at 21 weeks' gestation after thorough counseling. Her family history, young-onset disease, and histopathological findings suggested HBOC. She declined genetic testing for BRCA1/2, though genetic counseling was provided. In cases of pregnancy-related breast cancer, consideration must be given to whether the pregnancy should be continued and to posttreatment fertility. HBOC should also be considered. Genetic counseling should be provided and the patient should be checked for the* BRCA* mutation, as it is meaningful for the future of any potential children. Genetic counseling should be provided even if the cancer is advanced or recurrent.

## 1. Introduction

Pregnancy-associated breast cancer is a rare condition after cervical carcinoma [1]. However, it may be associated with hereditary breast and ovarian cancer (HBOC), because the diagnosis of breast cancer at a young age is associated with a higher likelihood of developing HBOC [2]. Furthermore, it is an indicator of HBOC in addition to the family history and tissue pathology. At an appropriate time, genetic counseling should be provided, and the patient with pregnancy-associated breast cancer should be checked for the BRCA mutation. Herein, we describe a case of recurrence of pregnancy-associated breast cancer that is suspected to be HBOC based on the family history and pathology of the isolated tissue.

## 2. Case Presentation

**Figure 1:**
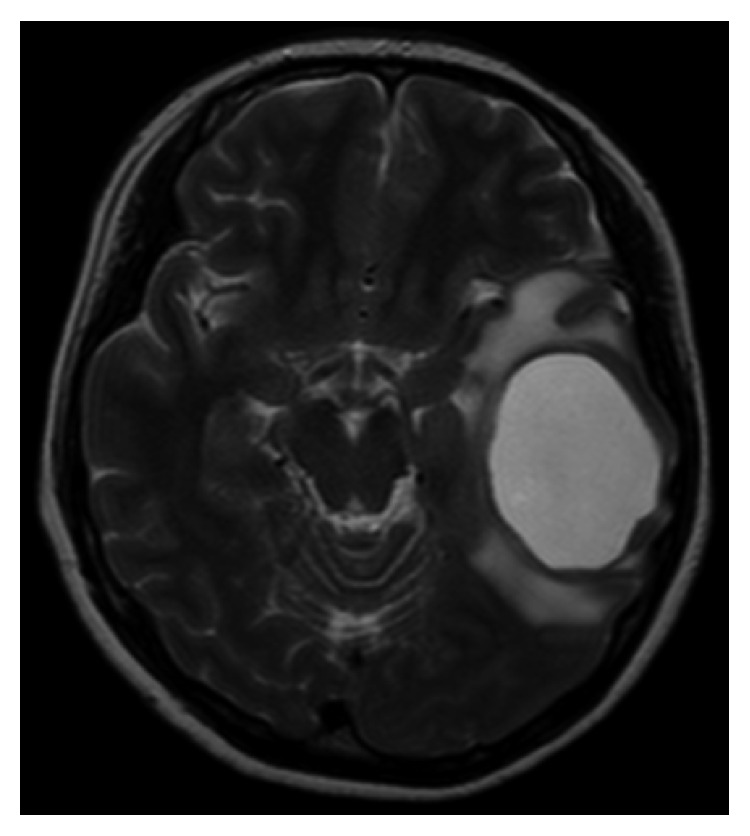
Brain magnetic resonance imaging (horizontal) indicating a solid mass in the left temporal lobe.

A 35-year-old pregnant Japanese woman (gravida 2, para 0-1-0-1) presented to our emergency outpatient department at 18 weeks' gestation with sudden dyslexia and worsening symptoms of aphasia and dyslexia. Brain magnetic resonance imaging (MRI) revealed a solid mass on the left temporal lobe (Figure 1).

**Figure 2:**
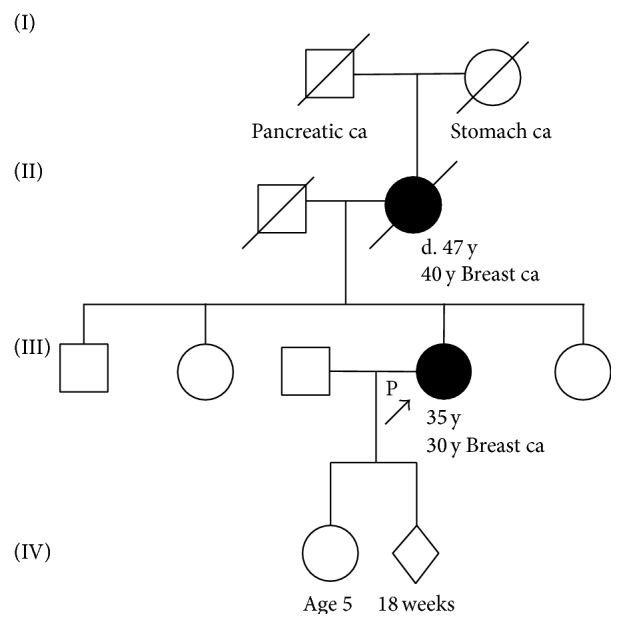
Pedigree of the patient's family. The cancer type or site followed by the age of diagnosis is indicated for pancreatic carcinoma (Pancreatic ca) and breast cancer (Breast ca) and stomach cancer (Stomach ca). The age in years or weeks (wks) at the time of ascertainment or age at death (d.), if known, is shown.

The patient's mother was diagnosed with breast cancer at 40 years of age and died as a result of the disease at 47 years of age; moreover, her grandmother also died of cancer (the affected organ was unclear) and her grandfather had pancreatic cancer. Her sisters and brother did not have cancer (Figure 2).

**Figure 3:**
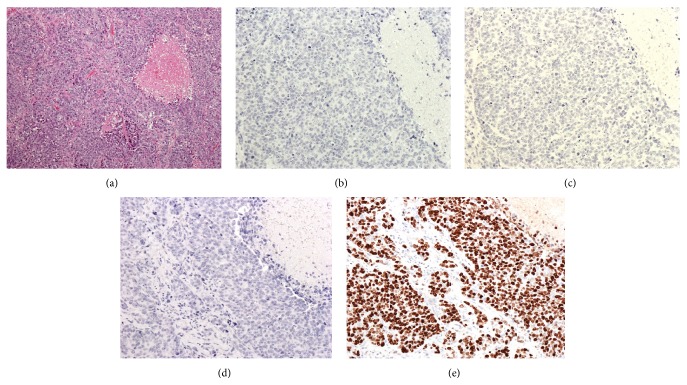
Histological examination indicates a diagnosis of invasive ductal carcinoma ((a) hematoxylin-eosin [HE] staining). Immunohistochemical examination indicates that the tumor was negative for human epidermal growth factor receptor 2 (HER-2) (b), estrogen receptor (ER) (c), and progesterone receptor (PR) (d). Moreover, the Ki-67 expression index is 80% (e).

At 29 years of age, she developed ovarian cysts and a salpingo-oophorectomy was performed. Subsequently, at 30 years of age, she was incidentally diagnosed with pregnancy-associated breast cancer during her first pregnancy; at that time, a cesarean section was performed at 36 weeks' gestation due to the termination of the pregnancy. Thereafter, right mastectomy and right axillary dissection were also performed. Computed tomography (CT), MRI, and bone marrow scintigraphy did not indicate any metastatic lesions, and the preoperative levels of tumor markers were as follows: NCC-ST-439, 22.5 U/mL (normal, ≤7.0 U/mL); CA15-3, 25.0 U/mL (normal, ≤27.0 U/mL); and CEA, 1.1 U/mL (normal, ≤5.0 U/mL). Furthermore, histopathological examination indicated that the tumor was an invasive ductal carcinoma. The tumor was located in the right breast and was 30 × 45 × 50 mm in size. Negative margins were obtained with surgery. No lymph node metastases were observed (0/14). The final pathological staging was T3, N0, M0, stage IIb. A hormone receptor assay indicated that the tumor was negative for both estrogen and progesterone receptors, and the human epidermal growth factor receptor 2 (HER-2)/neu test also yielded negative results. The Ki-67 expression index was 80% (Figure 3). Because she had triple-negative cancer, adjuvant therapy was recommended; however, she refused this therapy due to the risk of infertility. Four years after the surgery, no increases in tumor marker levels were noted. She became pregnant naturally after 4 years and came to our hospital for a prenatal checkup.

On admission, the patient's height was 160 cm and weight was 60.8 kg (body mass index, 38.0). The levels of tumor makers were within the normal ranges, as follows: NCC-ST-439, ≤2.5 U/mL (normal, ≤7.0 U/mL); CA15-3, 7.7 U/mL (normal, ≤27.0 U/mL); and CEA, 0.5 U/mL (normal, ≤5.0 U/mL).

**Figure 4:**
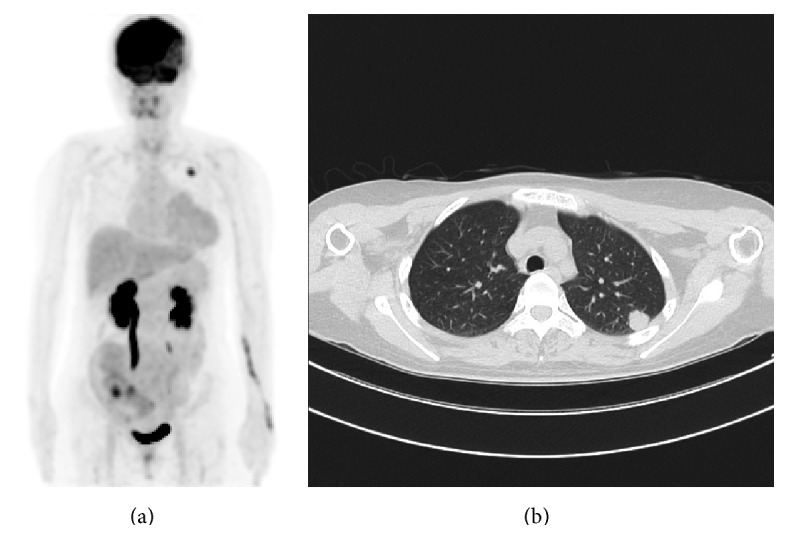
Positron emission tomography-computed tomography indicates the presence of lung metastasis at the left lung apex.

**Figure 5:**
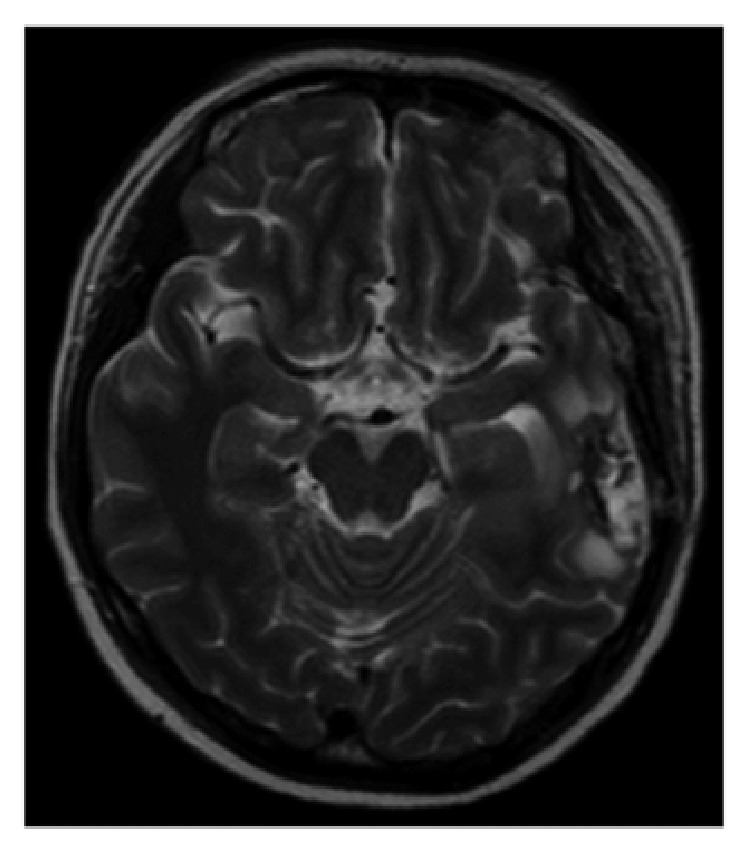
Brain magnetic resonance imaging (horizontal) indicating the absence of the metastatic tumor after urgent craniotomy and chemoradiation.

She underwent an urgent craniotomy. Histopathological examination of the tumor tissue indicated that the tumor was a metastasis of adenocarcinoma. The hormone receptor assay indicated that the tumor was negative for both the estrogen and progesterone receptors, and negative results were obtained on HER-2/neu testing. The Ki-67 expression index was 90%. To treat the recurrence of familial breast cancer during pregnancy, a multidisciplinary team including a breast surgeon, neurosurgeon, and radiotherapist discussed the case. To confirm whether there was metastasis at other locations, positron emission tomography-CT was also performed, and lung metastasis was detected (Figure 4). The breast surgeon believed that long-term survival is difficult in the case of brain metastasis, and, hence, treatment could proceed rapidly and she could continue her pregnancy while receiving anticancer medication until the date of delivery. However, the neurosurgeon and radiotherapist believed that she should undergo cranial irradiation after terminating the pregnancy. The patient and her husband did not wish to terminate the pregnancy, and, therefore, we determined a course of treatment after several consultations with the patient and her husband. However, they consented to terminating the pregnancy after the symptoms associated with the brain metastasis worsened. Therefore, an artificial abortion was performed at 21 weeks' gestation using gemeprost, after which whole brain irradiation was performed. Thereafter, she was treated with adjuvant chemotherapy containing adriamycin and cyclophosphamide and underwent a partial pneumonectomy. At present, she has not shown any signs of recurrence (Figure 5). Her family history, young-onset disease, and histopathological finding of triple-negative cancer were consistent with HBOC. Genetic counseling was performed. Though she understood the importance of a genetic test for her daughter, she did not wish to receive the genetic test. Her remaining ovary is regularly examined because she is at a high risk of developing HBOC.

## 3. Discussion

Pregnancy-associated breast cancer is the most common solid tumor in pregnancy after cervical carcinoma, but it is still rare. Pregnancy-associated breast cancer is defined as breast cancer that occurs during pregnancy or one year after delivery. It has been associated with a poor prognosis, though this is based on a limited number of retrospective case-control studies [3]. However, approximately 10–20% of breast cancer cases show familial clustering [4], and pregnancy-induced breast cancer could be HBOC because the patients are young at onset. Diagnosis of breast cancer at a young age is associated with a higher likelihood of developing HBOC [2]. In the early 1970s, Lynch et al. determined that familial breast cancer is an autosomal-dominant disease, and, in 1980, Lynch et al. defined familial breast cancer as the presence of the disease in at least two or more first-degree relatives [5]. Thereafter, in 1994, germline mutations in* BRCA1* were discovered and were thought to genetically predispose individuals to the disease. Lynch et al. have indicated that the genetic predispositions have been identified for approximately 40% of familial breast cancer families [6]. The common hereditary forms of breast cancer have been primarily attributed to the inheritance of mutations in the BRCA1 or BRCA2 genes [7]. However, not all families with hereditary breast cancer exhibit the* BRCA1* mutation; in fact, less than half of the families with site-specific breast cancer show an association with* BRCA1* mutations [8]. The National Comprehensive Cancer Network (NCCN) guidelines recommend that women aged ≤60 years with triple-negative breast cancer should be referred for genetic counseling [9]. The terms triple-negative and basal-like breast cancer (*BRCA1* genotype) have been used interchangeably [10], because breast cancer with a* BRCA1* genotype represents a basal tumor subtype [11] (Table 1). The triple-negative subtype of breast cancer accounts for 10% of all cases of breast cancer. For women with a significant family history of breast cancer, genetic testing of* BRCA1/2* is available as a routine clinical test for the diagnosis of HBOC in the US and other western countries [12]. In the present case, we strongly recommended radiation therapy or chemotherapy after the initial surgery, but the patient refused because she was worried that it would reduce her fertility. Since genetic counseling would not directly affect her prognosis, we did not recommend it. Genetic counseling was provided when treatment for the recurrent tumor ended because the patient had a 5-year-old child. The patient was amenable to genetic testing, but, partially due to its high cost, she did not consent to the test.

Genetic testing does not directly affect a patient's prognosis, but finding a* BRCA* mutation would be useful information for the patient's descendants. Children with a family history of breast cancer may live with vague worries of developing cancer given the familial aggregation. However, if the parent's* BRCA* mutation status is known, future genetic tests could check whether the child carries the mutation. Because only the gene containing the mother's mutation would need to be examined, the testing would likely be less expensive. If the child is negative for the mutation, her anxiety would be alleviated and she would only have to undergo regular breast cancer screening. If the type of HBOC mutation is known, the child would have the proper knowledge to undergo surveillance as a HBOC mutation carrier. In 2006, the American Society of Clinical Oncology and the Society of Surgical Oncology described the surgical management strategy for hereditary cancer syndromes [13]. The surgical risk-reducing treatment of hereditary cancer is associated with a varying risk of mortality [14]. Moreover, carriers of the* BRCA* mutation may use tamoxifen for breast cancer prevention or treatment. Furthermore, hormone replacement therapy is being more often prescribed after surgical menopause, and oral contraceptives are also recommended for ovarian cancer prevention [15].

Previously, adjuvant therapy was not considered after surgery due to the risks of infertility. However, there have been remarkable advances in the treatment of breast cancer and reproductive medicine. Cryostorage of ovarian tissue for fertility preservation is a new option, wherein ovarian tissue is removed prior to cancer treatment. In cases with triple-negative breast cancer, combined therapy with surgery and chemoradiation can be recommended, because the effect of internal secretion therapy and antiHER2 therapy is not sufficient, and a poor prognosis and early recurrence may occur [16]. In 2013, the Japan Society for Reproductive Medicine published a guideline for cryopreservation in cancer patients who would like to become pregnant in the future. However, there is a risk of spontaneous recurrence due to the transplantation of ovarian tissue. Ernst reported a case wherein a woman became pregnant following transplantation of frozen/thawed ovarian tissue but then underwent a legal abortion due to the recurrence of breast cancer [17]. The latest information on breast cancer should be made available to obstetricians, as this knowledge is linked to the field of obstetrics and gynecology.

We experienced a case of pregnancy-related breast cancer that metastasized to the brain, which we suspected to be HBOC. Although pregnancy-related breast cancers are often advanced, there is still value in providing genetic counseling and genetic testing at an appropriate time.

## Figures and Tables

**Table 1:** Surrogate definitions of the intrinsic subtypes of breast cancer.

Intrinsic subtype	Clinicopathologic definition
Luminal A	*Luminal A*
	ER- and/or PgR-positive, HER2-negative, and low Ki-67 expression

Luminal B	*Luminal B (HER2-negative)*
	ER- and/or PgR-positive, HER2-negative, and high Ki-67 expression
	*Luminal B (HER2-positive)*
	ER- and/or PgR-positive, amplified or overexpression of HER2, and high/low Ki-67 expression

Erb-B2 overexpression	*HER2-positive (nonluminal)*
	ER- and PgR-negative and amplified or overexpression of HER2

Basal-like	*Triple-negative (ductal)*
	ER- and PgR-negative and HER2-negative

ER: estrogen receptor; PgR: progesterone receptor.
